# A multi-scale evaluation of pack stock effects on subalpine meadow plant communities in the Sierra Nevada

**DOI:** 10.1371/journal.pone.0178536

**Published:** 2017-06-13

**Authors:** Steven R. Lee, Eric L. Berlow, Steven M. Ostoja, Matthew L. Brooks, Alexandre Génin, John R. Matchett, Stephen C. Hart

**Affiliations:** 1 U. S. Geological Survey, Western Ecological Research Center, Yosemite Field Station, Oakhurst, California, United States of America; 2 Vibrant Data Labs, San Francisco, California, United States of America; 3 U. S. D. A. California Climate Hub, Agricultural Research Service, John Muir Institute of the Environment, University of California Davis, Davis, California, United States of America; 4 Institut des Sciences de l’Evolution, CNRS, Université de Montpellier II, Montpellier, France; 5 Life and Environmental Sciences and Sierra Nevada Research Institute, University of California, Merced, California, United States of America; National University of Mongolia, MONGOLIA

## Abstract

We evaluated the influence of pack stock (i.e., horse and mule) use on meadow plant communities in Sequoia and Yosemite National Parks in the Sierra Nevada of California. Meadows were sampled to account for inherent variability across multiple scales by: 1) controlling for among-meadow variability by using remotely sensed hydro-climatic and geospatial data to pair stock use meadows with similar non-stock (reference) sites, 2) accounting for within-meadow variation in the local hydrology using in-situ soil moisture readings, and 3) incorporating variation in stock use intensity by sampling across the entire available gradient of pack stock use. Increased cover of bare ground was detected only within “dry” meadow areas at the two most heavily used pack stock meadows (maximum animals per night per hectare). There was no difference in plant community composition for any level of soil moisture or pack stock use. Increased local-scale spatial variability in plant community composition (species dispersion) was detected in “wet” meadow areas at the two most heavily used meadows. These results suggest that at the meadow scale, plant communities are generally resistant to the contemporary levels of recreational pack stock use. However, finer-scale within-meadow responses such as increased bare ground or spatial variability in the plant community can be a function of local-scale hydrological conditions. Wilderness managers can improve monitoring of disturbance in Sierra Nevada meadows by adopting multiple plant community indices while simultaneously considering local moisture regimes.

## Introduction

High elevation meadows are a vital ecological component of mountain systems throughout western North America. They provide critical habitat for wildlife [1, 2], supply key ecosystem services [3], and are favored destinations for people visiting the mountains. Understanding the extent to which human activities influence meadows is crucial for the preservation of these highly valued ecosystems. In the Sierra Nevada of California, pack stock (primarily horse and mule) use has become a major part of the conversation regarding wilderness conservation and management. Pack stock are used to carry people and supplies into remote sections of wilderness where pack stock are often turned out to forage, drink water, and rest in mountain meadows. Concerns about potential negative impacts from pack stock use have been voiced by various stakeholders and legal actions have been taken against some public land agencies responsible for the conservation and oversight of meadow habitats [4]. Highlighted in this discourse are specific concerns over whether pack stock are causing direct negative impacts to meadow plant communities through herbivory, trampling of vegetation, and soil disturbance [5, 6].

The biophysical characteristics of meadows in the Sierra Nevada are highly variable, especially related to hydrologic regimes and associated plant community types [7]. In the semi-arid landscape of the Sierra, water availability operates at multiple scales strongly influencing meadow plant community structure. Among meadows, variability in plant communities may be due to larger-scale influences on water availability such as elevation, regional climate, or basin hydrology [8], whereas within-meadow variability is largely an outcome of heterogeneity in local soil moisture regimes [9]. Complicating processes at each scale is the high inter-annual variability in moisture conditions that occurs across the Sierra Nevada [10]. Inter-annual variability in meadow moisture can have a strong influence on meadow vegetation that may outweigh disturbance impacts from pack stock. For example, Holmquist et al. [11] found moderate negative effects of pack stock use on coarse vegetation metrics (i.e., increased bare ground), yet these results were small relative to a strong effect of year (most likely from yearly differences in snowpack) on total vegetation cover in meadows. Similarly, wet versus dry meadows at two ends of the productivity spectrum differ greatly in hydrologic regime and plant community structure [7], and differences likely outweigh the more localized and potentially lesser effects of pack stock use [12]. In addition, different meadow types can display varied resilience to vegetation removal, such that some display compensatory growth and may increase in cover [12]. These considerations suggest that if pack stock do significantly affect meadow plant communities, detecting these effects will be difficult unless the underlying variability among meadow types is controlled for.

Here, we assessed meadow plant community responses to pack stock use while simultaneously controlling for multi-scale environmental factors known to influence variability of meadow hydrology and plant community structure. Specifically, we asked whether current levels (2004–2009) of pack stock use influence: total vegetation cover and bare ground, plant community composition, and local-scale spatial variability in plant community composition. We used a multi-step approach to: 1) control for large-scale, among-meadow variability by matching pack stock use meadows with non-pack stock reference meadows from a comprehensive database of remotely sensed estimates of hydro-climatic and geospatial attributes; 2) control for within-meadow variation in local hydrology by measuring in-situ soil moisture in all sampling plots and stratifying analyses by vegetation grouped to specific moisture regimes; and 3) control for variation in intensity of pack stock use by sampling across a large gradient in reported use.

## Methods

### Study area

**Fig 1:**
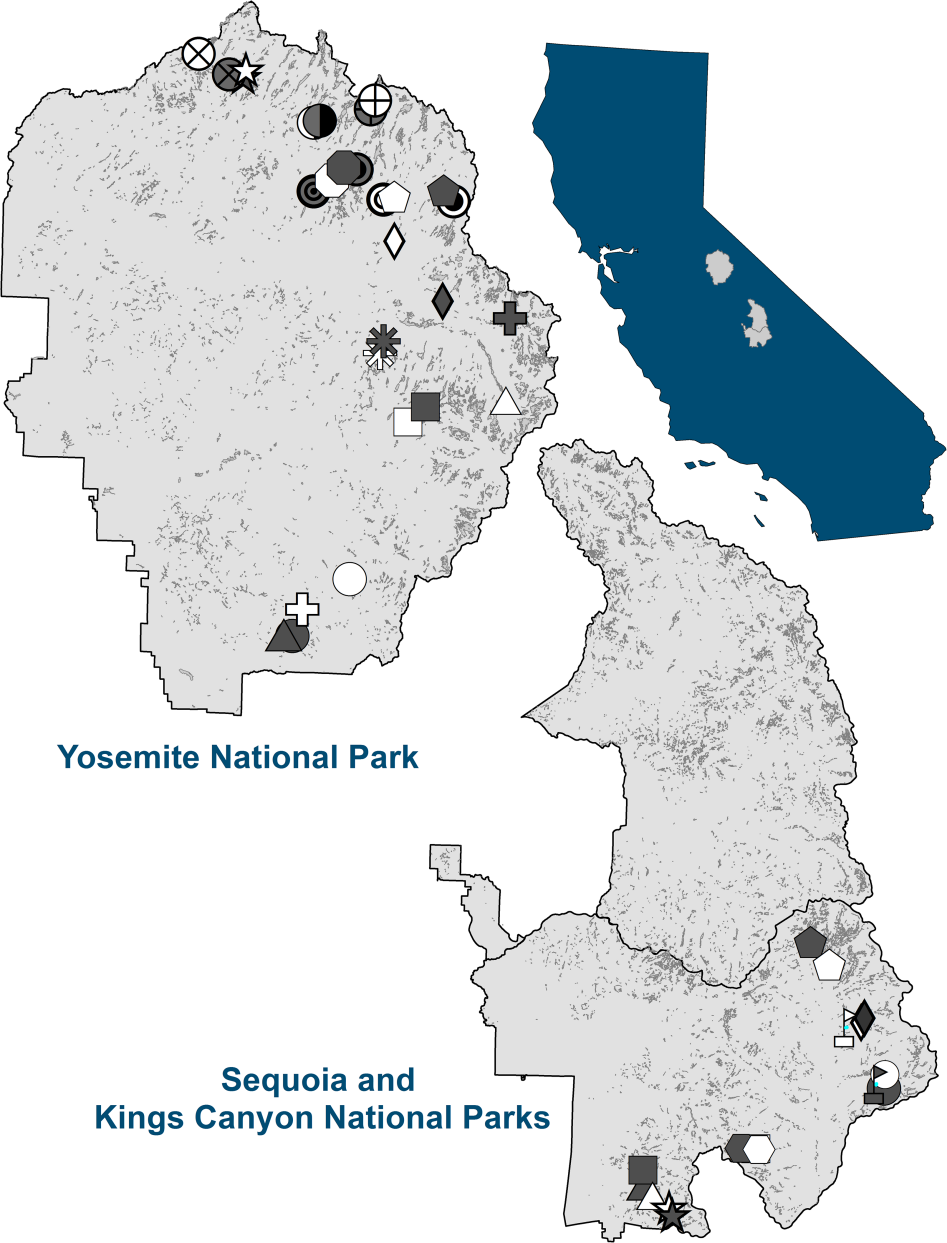
Map of study sites in Sequoia (SEKI) and Yosemite (YOSE) National Parks, California. Light gray polygons within each park represent mapped meadow units. The location of sampled meadow pairs are represented by matching symbols. White symbols are pack stock meadows while dark gray symbols are non-pack stock meadows.

The study was conducted in subalpine meadows within the Sequoia National Park section of the jointly managed Sequoia & Kings Canyon National Parks (SEKI) and Yosemite National Park (YOSE) in the Sierra Nevada, California (Fig 1, S1 Table). Research permits and approvals for fieldwork in both parks were obtained from the US National Park Service (NPS). The Sierra Nevada subalpine zone varies in elevation with respect to latitude, aspect, and local climate, but generally occurs between 2,450–3,600 m. Like much of the Sierra Nevada, soils in the subalpine zone are poorly developed (i.e., Entisols and Inceptisols), with most originating from granitic parent material that has received repeated glaciation during the recent Pleistocene epoch [13]. The zone can be described as a continuous complex of mixed conifer forests (predominately *Pinus contorta)*, rocky outcrops, and scrub vegetation types interspersed with highly diverse and productive meadow habitats [13]. The Sierra Nevada experiences a Mediterranean-type climate, with cool, wet winters (October-April) and a warm, dry summer season, with most of the water input to the subalpine and higher elevations falling as snow during the winter months. The growing season for the meadows varies with the timing of snowmelt, but typically runs from late May through August.

Subalpine meadows generally occur at perennially wet locations where ground water is at or near the surface [8], and plant species composition is closely linked to the underlying local soil hydrology [7,9]. Meadows in the region are dominated by perennial herbaceous plant species, including graminoids (e.g., *Carex scopulorum*, *Calamagrostis muiriana*) and forbs (e.g., *Oreostemma alpigenum*, *Gentiana newberryii*), interspersed with moss in the understory. Subalpine meadows are also used by native herbivores including mule deer (*Odocoileus hemionus californicus)*, voles (*Microtus* spp.), and pocket gophers (*Thomomys* spp.).

Pack stock use in Sierra Nevada meadows offers an opportunity to investigate the role of disturbance on plant communities at a scale relevant to management. Many of the meadows used by pack stock occur within National Parks, where meadows are free from confounding influences of other domestic livestock, such as cattle or sheep grazing. And unlike many cattle grazing practices, pack stock use within meadows occurs intermittently throughout the summer season as groups travel through [12]. Pack stock use also differs in terms of intensity. The number of individual animals allowed in a single group has been limited in YOSE and SEKI since 1972. Current numbers are set at 25 animals per group in YOSE and 20 animals per group in SEKI with some popular areas restricted to smaller groups [14, 15]. Total pack stock numbers within the National Parks have been declining since the 1960s [16] and are currently at historically low levels. Use levels for SEKI averaged 7,594 stock nights during the period of 1993–2002, followed by an average of 6,775 for 2002–2012 [17].

### Meadow selection

To understand if meadow plant communities were affected by stock use, we paired pack stock meadows to non-stock meadows using an ensemble of biophysical, geospatial, and hydro-climatic remote sensing data, which allowed us to compare sites that theoretically should have similar ecological conditions. Pack stock meadows were any meadow that received reported pack stock use within the past decade, whereas non-stock meadows had no reported pack stock use from any time period. The pool of potential meadows to select from was identified using park-wide meadow GIS layers [2]. Use records for each meadow were based on the number of "stock nights" (i.e., the number of pack animals times the number of nights using the meadow). All meadows with at least 10 reported stock nights during at least one of the most recent six years (2004–2009) of available data were considered as potential stock meadows. This allowed us to evaluate sites in the context of modern use levels. Stock meadows were then constrained by meadow size (< 25 ha) in order to: (1) allow for feasible sampling of multiple meadows within the short growing season, and (2) avoid uncertainty of stock use patterns in very large meadows where reported use does not identify locations where stock tend to graze or aggregate. Stock use records for candidate meadows were crosschecked to ensure accuracy by NPS ecologists in each park.

The twenty candidate stock meadows with highest number of stock nights from each park were matched to non-stock meadows, using the package ‘Matching’ [18] in the R programming environment (version 2.13.0 [19]). Matches were based on generalized Mahalanobis distances that take into account correlations among covariates for comparing groups (i.e., meadows) [20]. For each stock use meadow, one paired control meadow was selected from a potential pool that included all identified non-stock meadows within each park (3,606 in SEKI and 2,440 in YOSE) using remote sensing data and a multivariate matching technique. This matching process was designed to identify meadows with similar ecological characteristics so that the effects of pack stock on vegetation would not be obscured by the likely greater effects of variation among meadow types. Twenty-seven remotely sensed covariates were used for matching (Table 1), including vegetation indices that are directly related to meadow hydro-geomorphic types (e.g., Tasseled Cap Greenness) [7, 21]. We considered and rejected the possibility that these large-scale (averaged over entire meadows) indices may also detect pack stock effects on vegetation and therefore confound our study design. No such pack stock effects have been documented using these remote-sensing indices and we think this is because pack stock utilize only small portions of meadows that are much smaller than the resolution of the satellite-based remote sensing products used in this study. Variables related to meadow accessibility and potential use by hikers were also used in the matching process to avoid potential confounding effects.

**Table 1:** Twenty-seven geospatial, hydro-climatic, and vegetation variables derived from remote sensing used for multivariate matching of non-stock meadows to selected stock meadows in Yosemite (YOSE) and Sequoia (SEKI) National Parks.

**Variable**	**Description**
*Climate*	
Ranked Mean Precipitation*	Rank of meadow in mean precipitation (1980–2010) [22]
**Ranked Standard Deviation of Precipitation**	Rank of meadow in standard deviation of precipitation [22]
**Ranked Standard Deviation of Average Temperature**	Rank of meadow in standard deviation of Average Temperature [22]
Elevation*	Elevation at meadow centroid derived from 10-m Digital Elevation Models (DEM) [23]
*Hydrologic Regime*	
**Short Hair Sedge Cover (%)**	Percent of meadow polygon composed of vegetation alliance 7120—Short Hair Sedge [23]
**Semi-permanent Flooded Meadow Cover (%)**	Percent of meadow polygon composed of vegetation alliance 9000—Semi-permanent Flooded Meadow) [23]
**Ranked Mean Meadow Melt Date**	Rank of meadow in mean meadow melt data. Melt dates derived from MODIS snow cover data (2002–2007 [24]
**Ranked Standard Deviation for Meadow Melt Date**	Rank of meadow in melt data standard deviation [24]
**Ranked Standard Deviation for meadow 50% Snow Cover Date**	Rank of meadow in standard deviation of snow melt date when [24] meadow is 50% covered by snow
Ranked Mean Meadow 50% Snow Cover Date*	Rank of meadow in mean date for when meadow is 50% covered by snow [24]
Ranked Mean Tasseled Cap Greenness Index*	Rank of meadow from mean Tasseled Cap Greenness data Landsat-5 (1986–2006) 30-m resolution [25–27]
Ranked Standard Deviation for Tasseled Cap Greenness Index*	Rank of meadow from standard deviation of Tasseled Cap Greenness data from Landsat-5 30-m resolution [25–27]
**Ranked Average of Standard Deviation for Tasseled Cap Greenness**	Rank of meadow from the average standard deviation for Tasseled Cap Greenness data from Landsat-5 30-m resolution [25–27]
Ranked Mean Tasseled Cap Wetness*	Rank of meadow in mean Tasseled Cap Wetness 30-m resolution [25–27]
**Ranked Standard Deviation for Tasseled** Cap Wetness*	Rank of meadow in standard deviation of Tasseled Cap Wetness Landsat-5 30-m resolution [25–27]
**Ranked Average of Standard Deviation for Tasseled Cap Wetness**	Rank of meadow in standard deviation of Tasseled Cap Wetness Landsat-5 30-m resolution [25–27]
Ranked NDVI*	Rank of meadow in Normalized Difference Vegetation Index (NDVI) Landsat-5 30-m resolution [25–27]
Ranked Standard Deviation for NDVI*	Rank of meadow in standard deviation of NDVI 30-m resolution [25–27]
**Ranked Average of Standard Deviation for NDVI**	Rank of meadow in mean standard deviation of NDVI 30-m resolution [25–27]
*Accessibility*	
Distance to Nearest Lake *	Euclidian distance (m) to nearest lake [23]
Distance to Nearest Meadow*	Euclidian distance (m) to nearest meadow [23]
**Distance to Nearest Road**	Euclidian distance (m) of meadow to nearest road [23]
Distance to Nearest Trail*	Distance (m) to nearest trail [23]
**Estimated Minimum Travel Time From Trailhead**	Estimated travel time from trailhead (only used in YOSE) [23]
Nearest Meadow Cumulative Elevation Change*	Elevation change (m) between each meadow and nearest meadow [23]
**Nearest Meadow Maximum Slope**	Maximum slope between meadow and nearest meadow [23]
Meadow Area*	Hectares (ha) of individual meadow polygon [23]

* Variables used for calculating explanatory variables for Classification and Regression Tree Analysis (CART)

The three highest ranked matched reference meadows were visited and assessed qualitatively based on similarities in meadow size, landscape position (e.g., hill slope or basin), elevation and proximity with the matched stock meadow. The one deemed the best match was then selected as the non-stock (i.e., ‘control’) meadow for sampling. This resulted in every stock meadow having one sampled non-stock control meadow totaling to 22 matched pairs, with 14 pairs (14 stock/14 non-stock) sampled during the 2011 and 2012 growing seasons in YOSE and 8 pairs (8 stock/8 non-stock) sampled during the 2012 growing season in SEKI. The YOSE meadows ranged in size from 1.15–22.14 ha with an average meadow size of 5.90 ha, and SEKI meadows ranged in size from 0.42–10.62 ha with an average meadow size of 2.60 ha. The maximum number of stock nights in a single year varied widely during 2004–2009: 10–577 stock nights in YOSE, with an average of 134 per year; and 82–271 stock nights in SEKI, with an average of 155 per year.

### Field sampling

Each meadow was sampled once during the peak summer growing season (June–August). Sampling occurred along 5-m wide belt transects spaced 40 m apart, running across the meadow width, perpendicular to the main meadow drainage. Along the centerline of each belt transect, 2 x 2 m (4 m^2^) sampling plots were established at 20 m intervals. Ocular aerial estimates of the total vegetation cover (%), litter cover (%) and exposed mineral soil cover (i.e., % bare ground) were recorded within each plot. Plant species composition was sampled at every third sampling plot along each transect with ocular aerial estimates of cover by species in eight 25 x 25 cm sub-plots arranged systematically within each 4 m^2^ plot. Soil moisture was recorded at every 4 m^2^ plot as volumetric water content (VWC) using a Field Scout Time Domain Reflectometer 100 soil moisture probe (Spectrum Technologies, Plainfield, IL) to a depth of 12 cm. Readings were taken within 10 cm of the inside of each corner as well as the center of each plot. The soil VWC values were averaged within each plot.

### Data analyses

Classification and Regression Tree (CART) analyses were used (e.g., [28]) as a non-parametric approach for assessing the relative contribution of pack stock use versus other geospatial and hydro-climatic covariates (landscape level variables) in explaining differences in vegetation between paired stock and non-stock meadows. In order to utilize the paired study design, response and explanatory variables (described below) used in each CART analysis were calculated as the difference between each paired stock and non-stock meadow. This allowed for direct interpretation of each response in the context of pack stock use. CART was performed using JMP 10.0 (SAS Institute) and all other calculations were done using the statistical software R version 2.13 [19]. In cases where the CART analyses suggested an overall pack stock signal was present, we conducted more detailed assessments of individual response variables along gradients of pack stock use.

#### Response variables

Three metrics were used as response variables: bare ground, species dissimilarity and species dispersion:

1. Bare ground—Total cover of bare ground in each plot was used both as a direct measure of exposed soil and as an indirect measure of total vegetation cover. Bare ground and total vegetation cover (live and dead as litter) were negatively correlated (bare ground and vegetation cover, Pearson’s *r* = -0.65; and bare ground and vegetation plus litter cover, Pearson’s *r* = -0.84). We did not use vegetation or litter cover as response variables due to high Pearson’s r values with bare ground.
2. Species dissimilarity—Multivariate differences between stock and non-stock meadow plant communities were assessed by calculating a multivariate, Bray-Curtis dissimilarity distance between paired meadows. Distance matrices were based on species identity and untransformed cover values for individual plots, and computed using the VEGAN package in R [29]. Species dissimilarity measures overall differences in the identity and relative abundances of species between stock and non-stock meadows without taking into account any differences in the spatial patterning of the plant community.
3. Species dispersion—There is a possibility that plant communities can maintain overall similar species composition and relative abundances, but differ in the spatial patterning and variability of those species [30]. For example, trampling or grazing by stock could change the spatial scale of vegetation patchiness even if the community composition remains the same. We evaluated the patterns of local-scale spatial variability by calculating the multivariate “species dispersion” based on the identity and cover of species within each subplot for each individual 4 m^2^ sampling plot [31]. Species dispersion values were estimated by calculating the mean Bray-Curtis distance of each of the eight, 25 x 25 cm sub-plots to an ordinated plot centroid using the betadisper function in the VEGAN package [29]. A higher species dispersion indicates that the local vegetation community (4m^2^ scale) is more heterogeneous than a plot with lower species dispersion.

Mean soil moisture (VWC) measurements for each plot were used to stratify the analyses across distinct vegetation community types associated with a different dominant soil moisture regime (see Explanatory Variables below). In order to ensure an adequate sample size, those vegetation community types that had less than three plots for any meadow in a pair of matched meadows were dropped from the analysis. Differences in average bare ground and differences in mean species dispersions were calculated by subtracting the bootstrapped means of the non-stock (control) meadow from the bootstrapped means of the paired stock meadow. Each procedure was run 1000 times to produce an estimated mean difference as well as 95% confidence intervals for each meadow pair. These were then used as the response variables in each CART analysis. Mean estimates and 95% confidence intervals for species dissimilarity between matched pairs were calculated based on bootstrapped estimates of mean values for each species within each vegetation community type. Similar to the bare ground response, the procedure was run 1000 times and a median value from the resulting distribution was taken as a mean estimate to be used in the CART analysis.

#### Explanatory variables

Explanatory variables used in the CART analyses fell into three broad categories:

1. Pack Stock Use—Six measures of pack stock use based on NPS records from 2004–2009 were assigned to each matched pair: mean and maximum annual stock nights, mean and maximum annual stock density (i.e., stock nights per hectare), and the standard deviation and coefficient of variation of annual stock nights across the six year period. We considered this six year period as a representative snapshot of contemporary use levels.
2. Within-pair difference -The differences in physical attributes (e.g., size, elevation, hydrology, meadow melt date) within each stock and non-stock meadow pair were used to explain differences in vegetation between the pair. A large difference in each response due to Within-pair differences would suggest a weak pairing between stock and non-stock control meadows.
3. Between-pairs difference—The mean physical attributes of each meadow pair were used to evaluate how much the difference in vegetation between stock and non-stock meadows varies with the overall environmental context of the meadow pair (e.g., a high elevation pair might show a larger response than a low elevation pair).

Fourteen geospatial and hydro-climatic covariates derived from remote sensing data were used to measure within-pair and between-pairs differences (Table 1). We excluded covariates that were either highly correlated (r > |0.80|) or ones that exhibited extremely low variation among the selected meadows (e.g., percent cover of the Short Hair Sedge vegetation alliance, Table 1). For the CART analyses, to avoid model over-fitting and potential problems of interpreting model coefficients, trees were pruned to include only splits that added more than 10% to the total R^2^ and that did not have ties in the covariates selected for the split. We used a bootstrapping method to quantify uncertainty in the estimates of meadow-scale differences between stock and non-stock meadows of each pair to better describe the specific relationships between stock use intensity and meadow responses (described under Response Variables above).

**Fig 2:**
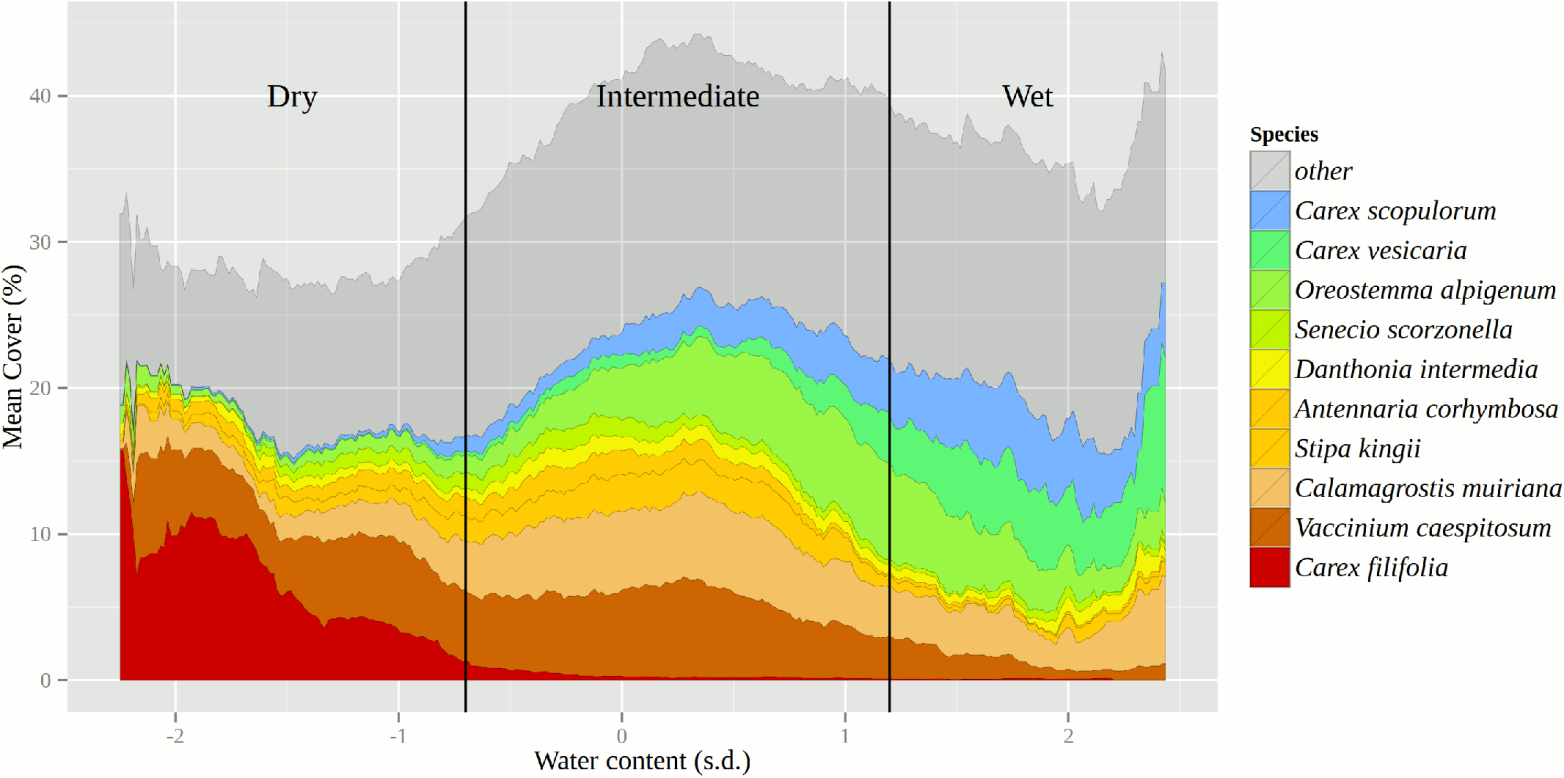
Moving averages of cover (%) for the 10 most dominant plant species along a gradient of soil volumetric water content (Water content) across all species plots (n = 1322). Shaded background represents moving average cover for all species present. Soil water content values are expressed as standard deviations (s.d.) from individual meadow means. Values run from driest plots on left (-3.0) to wettest plots on right (3.0). The moving average window width = 0.25 s.d. Vertical lines indicate breaks used to delineate individual vegetation community types (Dry, Intermediate, and Wet).

Natural variability in soil moisture regimes known to influence coarse scale patterns of dominant vegetation within each meadow were addressed by stratifying all analysis across three vegetation community types reflective of local soil moisture conditions (“Wet”, “Intermediate”, and “Dry”). To do this, patterns in the distribution of the 10 most dominant plant species were explored by plotting the mean plot level cover (computed as a running average) along the soil moisture gradient, taken as the mean plot level soil VWC standardized to individual meadow means. The moving average was calculated as a two-sided mean with a window width of 0.25 standard deviations. Vegetation community types were then delineated based on natural breaks and transitions in the distribution of species representative of dominant soil moisture regimes (see Results; Fig 2).

## Results

### Vegetation community types

The top 10 most abundant species showed clear vegetation community changes along the soil moisture gradient (Fig 2). These distributions guided the delineations made for Dry, Intermediate, and Wet vegetation community types used in the subsequent analyses. Dry vegetation communities had an average of 10.0 species per plot and were dominated by *Carex filifolia*, a xeric vegetation type indicator species [7]. *Carex vesicaria* and *Carex scopulorom*, both characteristic of wet meadow habitats [7], were highly abundant within the Wet vegetation community type, yet steadily decreased in abundance as plots transitioned into the Intermediate vegetation community. The Wet vegetation community type had an average of 11.8 species per plot. The Intermediate vegetation community type showed the greatest cumulative cover and supported the greatest species richness with an average of 12.1 species per plot.

### Landscape variables and pack stock

**Fig 3:**
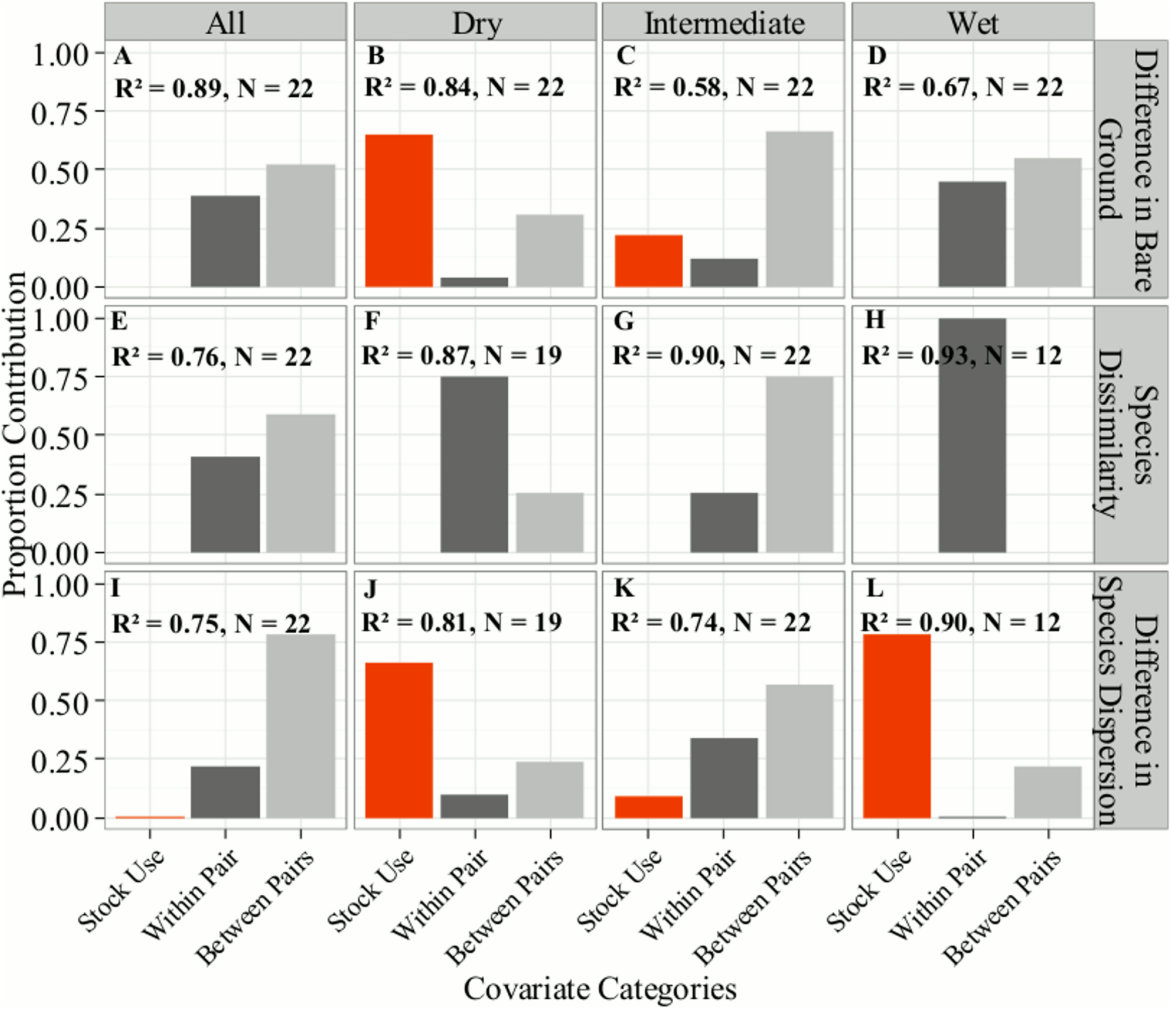
The relative contribution of various classes of covariates in explaining the differences between stock and non-stock meadow pairs for Classification and Regression Tree (CART) models. Separate models were run for each vegetation community type (Dry, Intermediate, and Wet) as well as the entire meadow scale (All). Each panel shows the CART R^2^ and sample size of meadow pairs (N). Covariate categories [shades]: Red (Stock Use) = Six measures of stock use for the grazed meadow of the pair; Medium gray (Within-pair) = differences in physical attributes between meadows within each stock and non-stock pair; Light gray (Between-pairs) = differences among meadow pairs in their mean physical attributes (see text).

Individual CART models were able to explain 58 to 93% of the variance in the difference between stock and non-stock meadows. However, the relative contributions of different classes of explanatory variables (e.g., Within-pair vs. Between-pairs) differed among responses (bare ground, species dissimilarity, and species dispersion) and by vegetation community type (Fig 3). At the whole meadow-scale, signals from pack stock use were weak to non-existent when compared to the landscape-scale hydro-climatic, and geospatial variables acting within (Within-pair) and among the meadow pairs (Between-pairs; Fig 3, Panels A, E, and I). Among these physical attributes, differences between the meadow pairs (Between-pairs) explained more variation in each response than any differences within a single pair at the whole meadow scale (Within-pair; Fig 3, light gray vs. dark gray bars).

One third of cases showed a significant signal of stock use on vegetation differences among meadows when analyses were stratified by individual community types (Table 2). These instances occurred with bare ground within the Dry vegetation community (Fig 3, Panel B) and species dispersion within the Dry and Wet vegetation communities (Fig 3, Panels J, L). For bare ground in the Dry vegetation community, maximum stock nights per hectare was the most important explanatory variable, with a 63% model contribution (i.e., proportion of total sum of squares). For species dispersion, maximum stock nights per hectare had 66% model contribution for the Dry vegetation community, while the standard deviation in stock nights had 78% model contribution for the Wet vegetation community. Stock Use did not explain any differences in overall multivariate species composition and abundance without consideration of spatial patterning (species dissimilarity; Fig 3, Panels E, G, H).

**Table 2:** Classification and Regression Tree (CART) model contributions (%) of specific stock use metrics. Individual models are indicated by vegetation community types and response variable combinations. Vegetation Community types: A = All; D = Dry; I = Intermediate; W = Wet. Instances where a Stock Use Metric contributed to more than 50% of a CART model are italicized.

Response Variable												
	Difference in bare ground				Species dissimilarity				Difference in species dispersion			
	Community Type				Community Type				Community Type			
	A	D	I	W	A	D	I	W	A	D	I	W
**Mean Stock Nights**	0	2	0	0	0	0	0	0	0	0	0	0
**Max. Stock Nights**	0	0	0	0	0	0	0	0	0	0	0	0
**Std. Dev. Stock Nights**	0	0	0	0	0	0	0	0	0	0	0	*78*
**C.V. Stock Night**	0	0	22	0	0	0	0	0	0	0	9	0
**Mean Stock Nights / ha**	0	0	0	0	0	0	0	0	0	0	0	0
**Max. Stock Nights / ha**	0	*63*	0	0	0	0	0	0	0	*66*	0	0

Std. Dev. = standard deviation; C.V. = coefficient of variation

### Gradients of pack stock use

The previous analysis contrasts the relative contributions of pack stock use and a suite of coarse physical meadow attributes for explaining differences between stock and non-stock meadow pairs. We now evaluate the three cases where splits in the CART models were contributed to more by a Stock Use metric than any of the mean physical attributes. For each example, the individual bootstrapped differences between meadows were plotted against the most contributing Stock Use metric (Table 2).

#### Bare ground

**Fig 4:**
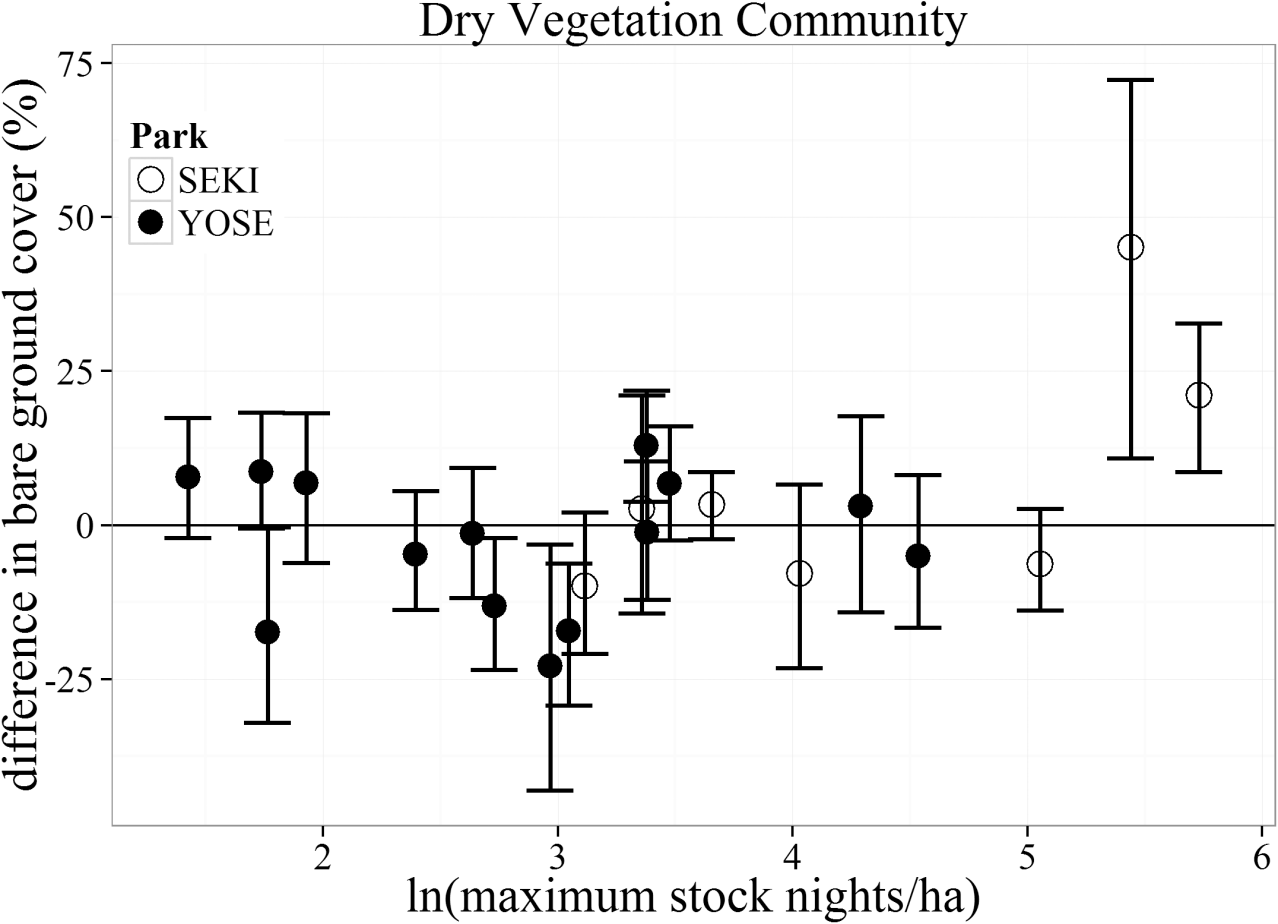
Bootstrapped mean (with 95% confidence intervals) differences in percent bare ground cover for the Dry vegetation communities between matched meadows (N = 22) in Yosemite (YOSE) and Sequoia (SEKI) National Parks. The range of 1–5.7 (ln) maximum stock nights/ha corresponds to a range of 4–308 untransformed maximum stock nights/ha.

Differences in bare ground cover within the Dry vegetation community type varied considerably across the entire range of maximum stock nights (Fig 4). Four meadows pairs ranging from low to moderate stock use intensities (maximum stock nights/ha) had greater bare ground in the non-stock meadow than the paired stock meadow (indicated by a negative value). Difference in bare ground was only significantly greater (indicated by a positive value) for the two highest stock use intensity meadows (230 and 308 maximum stock nights/ha), both of which were located in SEKI.

#### Species dispersion

**Fig 5:**
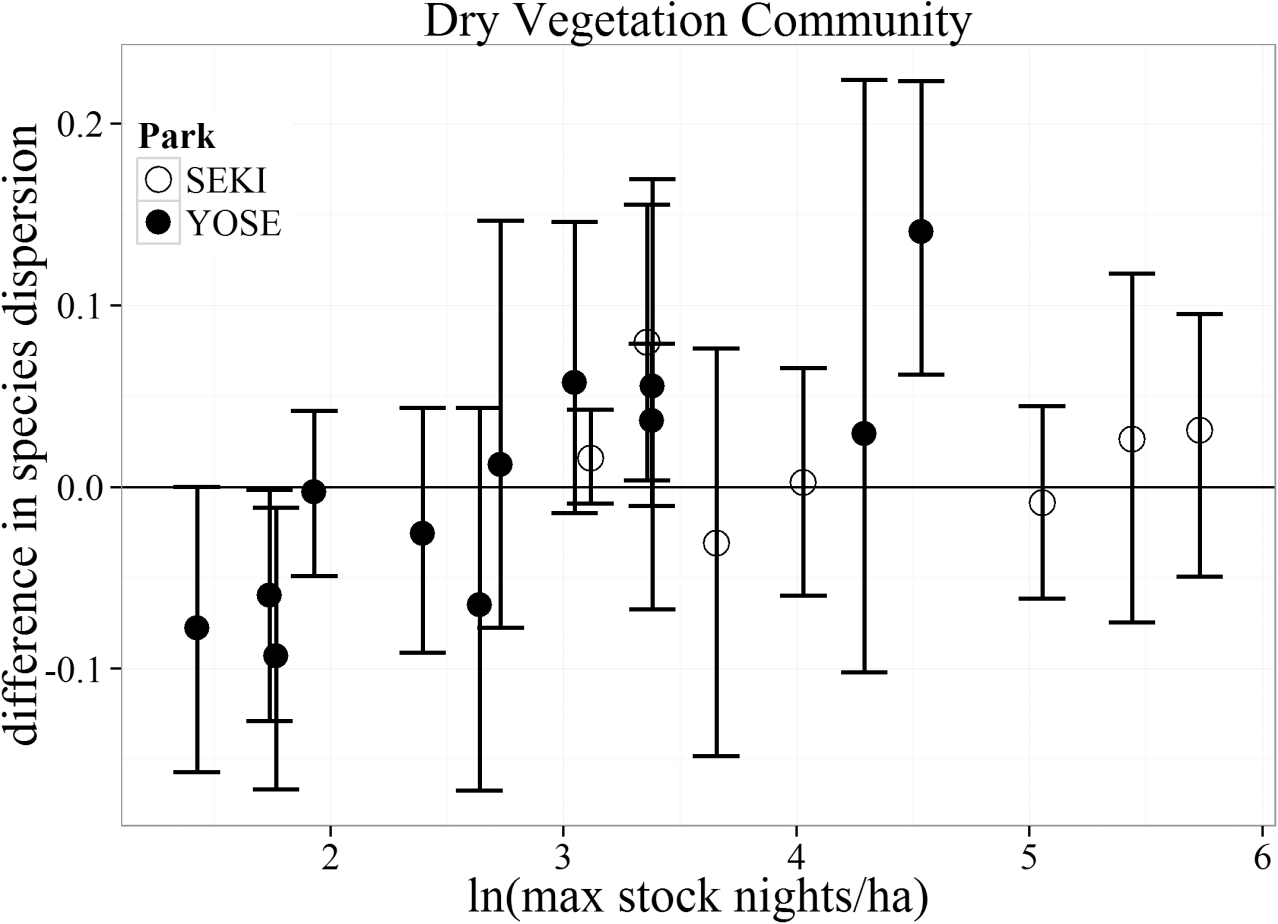
Bootstrapped mean (with 95% confidence intervals) differences in species dispersion for the Dry vegetation community between matched meadows (N = 19) in Yosemite (YOSE) and Sequoia (SEKI) National Parks. The range of 1–5.7 (ln) maximum stock nights/ha corresponds to a range of 4–308 untransformed maximum stock nights/ha.

**Fig 6:**
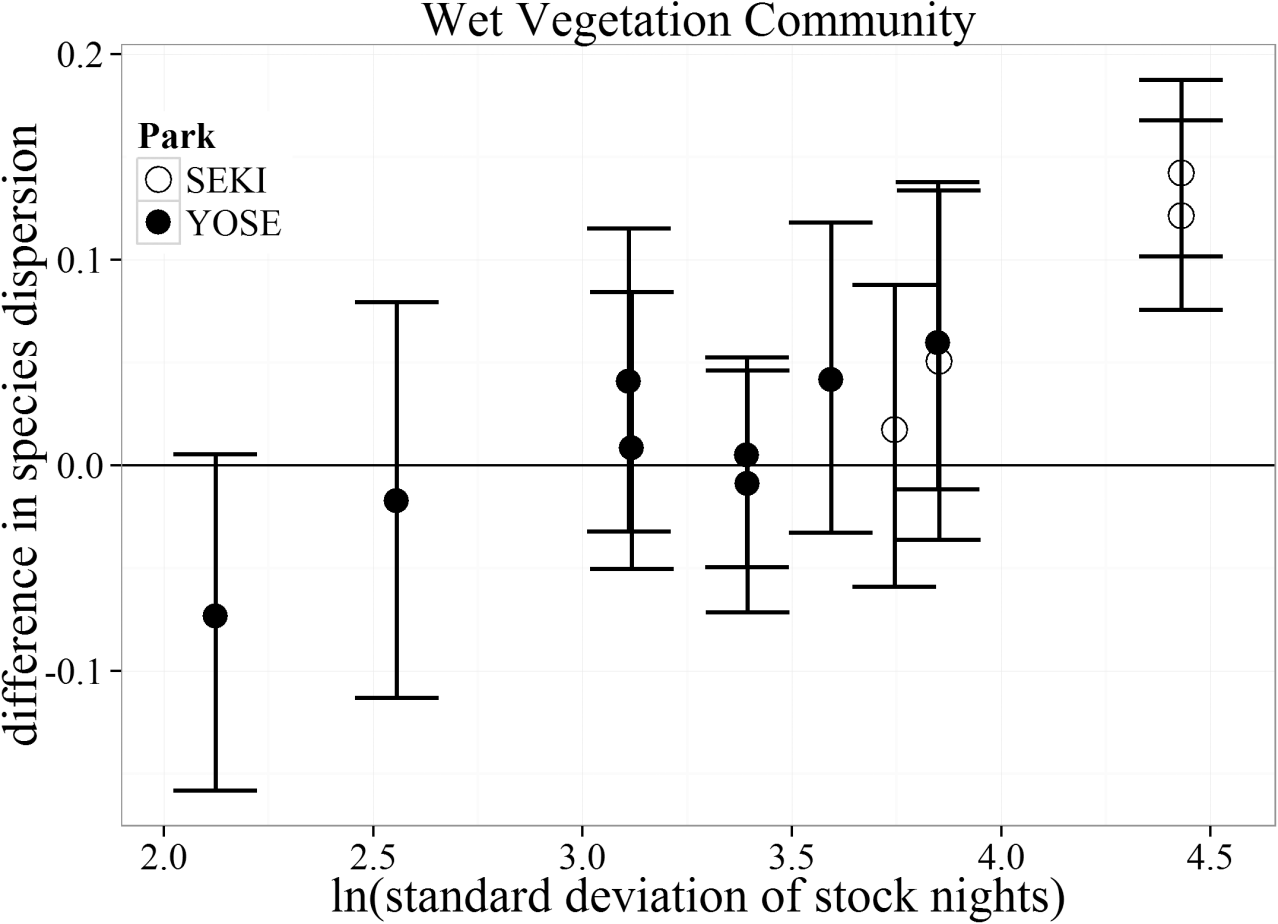
Bootstrapped mean (with 95% confidence intervals) differences in species dispersion for the Wet vegetation community type between matched meadows (N = 12) in Yosemite (YOSE) and Sequoia (SEKI) National Parks. The range of 1.4–4.9 (ln) standard deviation of stock nights corresponds to a range of 4–135 untransformed stock nights.

In general, there was little difference in local-scale spatial variability in plant community composition (species dispersion) between stock and non-stock meadows across all stock use intensities for both the Wet and Dry vegetation communities (Fig 5, Fig 6). The Dry vegetation community type showed greater species dispersion in non-stock than stock meadows at the two lowest stock use intensities (negative values, Fig 4). The only meadow pair within the Dry vegetation community type to show greater species dispersion within the stock meadow occurred at a moderate stock use intensity level. A much different pattern emerged within the Wet vegetation community. The top two meadows with the highest standard deviation in stock nights showed a greater species dispersion for stock use meadows than paired non-stock meadows (Fig 6). Similar to the trends for bare ground within the Dry vegetation community, these meadows were both located within SEKI. No trends of increased species dispersion were observed in YOSE.

## Discussion

Sierra Nevada meadows are complex ecosystems that are routinely subjected to natural disturbances such as seasonal flooding from snowmelt [32], long-term decadal droughts [33], and bioturbidation from small mammals [34]. Meadow plant communities, along with the environmental variables that help structure those communities, vary greatly within and across individual meadows, watersheds, and elevations. Thus, at the whole-meadow scale, it is not surprising that potential signals of pack stock use on meadow vegetation were swamped by these larger scale environmental factors. It was only by adopting a multi-scale approach that accounted for environmental processes known to influence variation in plant communities both among and within meadows that we were able to detect a meaningful “ecological signal” of pack stock use. Landscape- and regional-scale environmental variables were addressed through multivariate pairing of non-stock control meadows using remotely sensed data. Within-meadow scale variability was addressed by stratifying the analysis within meadows across vegetation community types (Wet, Intermediate, Dry) linked to the soil moisture gradient, which has strong influence on plant community structure [9]. Variation in pack stock use was controlled for by sampling across a large gradient of reported use and evaluating multiple metrics of pack stock use intensity. This multifaceted approach allowed for direct comparison of stock and non-stock meadow communities while controlling for variation in both the environmental and anthropogenic drivers. Results from our initial CART analysis showed that differences in the mean physical attributes between the meadow pairs (Between-pairs) explained more variation for each response than any differences within a single pair (Within-pair; Fig 3A, 3E and 3F). This result suggests that the overall pairing of stock with non-stock “control” meadows was successful at minimizing other sources of natural variation among meadows.

For bare ground cover, signs of potential impacts were restricted to the Dry vegetation community type. This suggests that current use levels may be having little impact on bare ground or vegetation cover across the majority of meadow plant communities (Wet and Intermediate). Furthermore, bare ground within the Dry vegetation community exhibited a non-linear relationship with pack stock, where only meadows with the highest use intensities (> 156 maximum stock nights/ha) had greater bare ground than their matched control meadows (Fig 4). Such a non-linearity suggests the possibility of a threshold response to pack stock use. For any meadow plant community type, there may be a point where increased pack stock use would result in increased bare ground and decreased vegetation cover. This threshold may be lower for the Dry vegetation community than for the other communities. Dry plant communities have inherently less total vegetation cover, slower growth rates, and are relatively less productive than wetter regions within the meadow [35]. This lower productivity could translate to lower resistance (ability to remain unchanged) or resilience (rate of return to a previous undisturbed state) to trampling and grazing by stock animals, especially if a threshold level has been reached [36]. This finding has direct management implications and warrants future work within dry meadow areas. If pack stock congregate within dry meadow communities (e.g., near pack stock camps) those areas may show larger reductions in vegetation cover than other parts of the meadow. Similar reductions in vegetation cover have been found in semi-arid systems at locations where wild horses tend to congregate (e.g., springs [37]).

A contrasting, yet equally important result, was the lack of any observed difference (stock vs. non-stock) in bare ground within the Intermediate or Wet vegetation communities. Meadow soils within these community types stay consistently moist throughout the growing season making them more susceptible to compaction due to lower soil strengths [38]. Soil compaction directly alters the physical structure of soil, creating a potential for slowed vegetation growth and increased exposure of bare mineral soil [39–41]. Yet, both of these community types support a much higher total vegetation cover as well as a greater plant species richness. These characteristics could contribute to a higher resistance to current pack stock use levels. Similar patterns have been observed in other grazed systems where areas with high species richness tend to display more temporal stability in biomass production in the face of ecological stressors [42]. Much of the work assessing potential relationships between grazing intensities and bare ground in riparian ecosystems has focused on trampling effects by cattle [36, 43]. Ecological thresholds crossed by high-density cattle grazing may be less commonly crossed by lower intensity and potentially more dispersed pack stock use [12].

**Fig 7:**
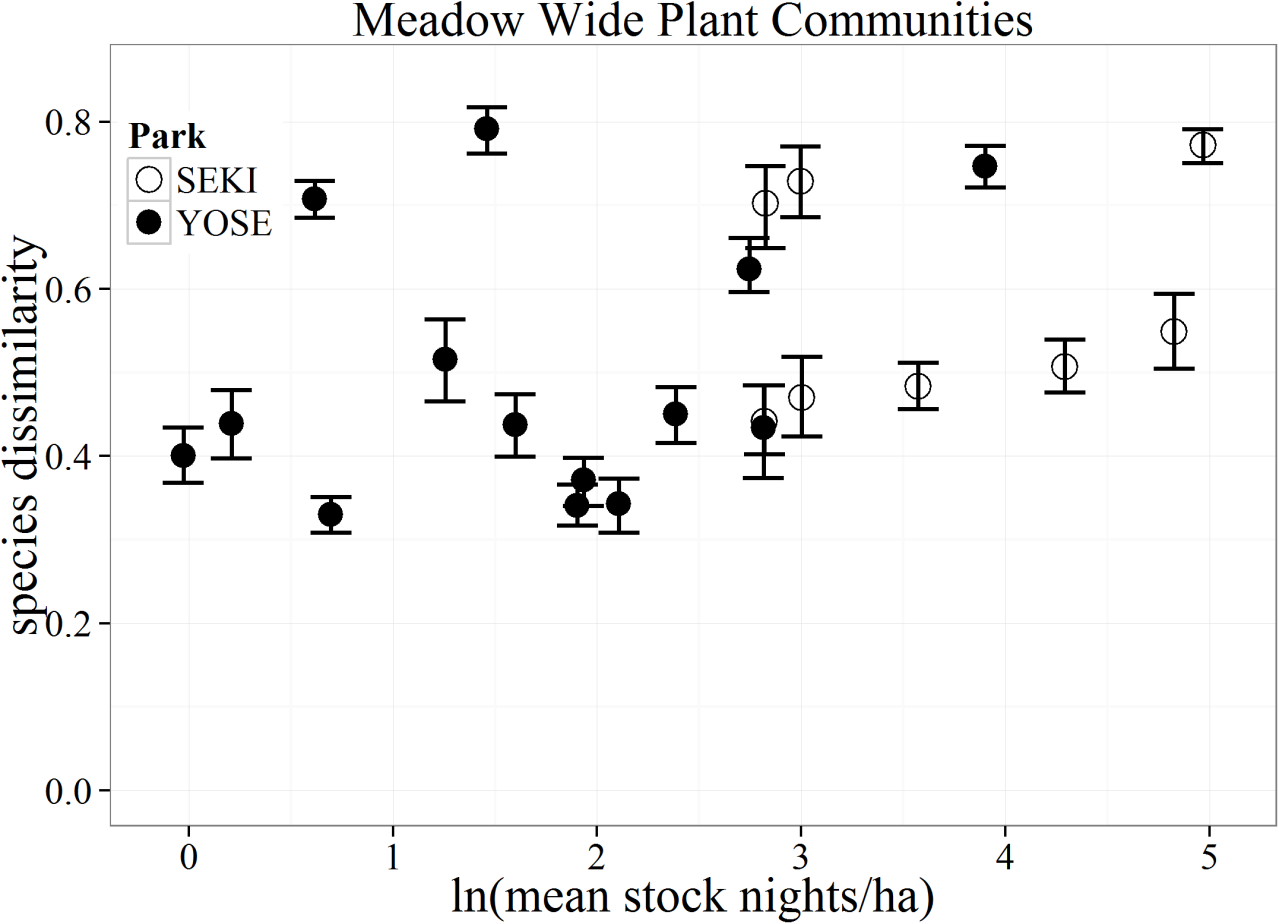
Bootstrapped mean (with 95% confidence intervals) differences in species composition (as Bray-Curtis dissimilarity distances) for all vegetation communities between matched stock and non-stock meadows (N = 22) in Yosemite (YOSE) and Sequoia (SEKI) National Parks.

Differences in the mean community composition (species dissimilarity) between paired stock and non-stock meadows was highly variable and showed no clear or consistent trend with stock use for any of the vegetation community types. Instead, results from the CART analysis suggest that hydro-climatic and geospatial attributes of meadow pairs were often the best predictors of large differences between stock and non-stock meadows. Our results support findings from NPS monitoring in SEKI where, over a 25-year monitoring period, meadows showed greater differences across meadow pairs than between individual paired stock and non-stock meadows [16]. Closer examination of meadow pairs showed highly variable differences in overall plant community structure across the entire gradient of pack stock use for all vegetation community types (Fig 7). If there was a strong relationship with pack stock use, we would have expected to see increasing divergence in the Bray-Curtis distances (dissimilarity) between our matched meadows with increasing pack stock use. Instead, meadow pairs with the greatest differences in composition were at relatively light-use levels. These pairs could represent meadow communities that are more sensitive than others to even light use, or may simply reflect variation in environmental variables other than pack stock use.

Our results suggest that, under certain conditions, variability in plant community composition (as species dispersion) could be a more sensitive response to ecological stressors than mean metrics of vegetation cover or species composition often used in monitoring grazing practices[44–46]. Similar measures of variability have been shown to be early warning sign of stress in other ecosystems (e.g., cattle grazing in arid environments [47]) and may relate to ecosystem stability and transitioning states [48]. The CART analyses we performed indicate that pack stock use is potentially influencing species dispersion in both the Wet and Dry vegetation communities; however, no effect was seen on Intermediate plant communities.

A closer examination of the vegetation community types shows two very different patterns of variability across our study meadows. Dry vegetation communities exhibited lower species dispersion (increased homogenization) within meadows with the lightest use (< 8 maximum stock nights/ha). Decreased spatial variability in plant communities experiencing grazing disturbance is not a novel concept [30]. Yet the scale and intensity of the disturbance mechanism in existing literature is very large (e.g., sustained herds of bison [49]) compared to the impacts we are investigating. With our results showing only marginally lowered species dispersion in the Dry community following pack stock use, we cannot confidently conclude a link to disturbance from pack stock. However, results from the Wet vegetation community show a much different pattern. Wet meadow plant communities exhibited greater species dispersion at the highest levels of stock use. This contrasts with the results discussed earlier where mean species dissimilarity showed no definitive changed due to stock use within the Wet vegetation community. The pattern of increased species dispersion might be conditional on interactions with physical attributes that characterize wet soils. Increased species dispersion could result when wet regions of a meadow remain saturated longer into the growing season; this condition may make these areas more susceptible to increased heterogeneity in micro-topography due to localized zones of compacted soils from trampling, which could lead to increased species dispersion [38, 50].

We expected that increased species dispersion would be extended into the Intermediate vegetation community because those areas tend to host more species, and therefore a greater potential for spatial variability among the species present. The failure to detect changes in species dispersion within the Intermediate vegetation community may be a matter of the spatial resolution sampled. Intermediate communities occupy a larger proportion of total meadow area compared to the Wet and Dry vegetation communities. As a result, this community type contains steep hydrologic gradients [51] with many different functional and hydrogeomorphic types of species [7], where strong biotic interactions (i.e., competition between high density species [52]) may be adding to the dynamic properties of the zone. The high levels of natural variability occurring due to these multiple processes could be obscuring effects that become observable at finer spatial resolutions.

The overall transition of the plant communities along the soil moisture gradient (Fig 2) reflected the close relationship plant communities have with soil water availability within meadows. Regardless of disturbance from pack stock, longer-term temporal changes in the soil moisture regime could have drastic changes on meadow plant communities. There is growing evidence that with climate change there may be an increased risk of drought for California [53]. Drought stress can open meadow plant communities up to invasion by more drought tolerant species [54], and disturbance from pack stock may exacerbate drought impacts. We did not detect signs of such interactions during our study (2011–2012), but prolonged drought such as that experienced in the Sierra during 2012–2015 [55] may have increased the susceptibility of these meadows to other plant stressors like pack stock disturbance.

Compositionally, we observed no differences along the entire pack stock use gradient sampled. Instead, we noticed differences at the highest pack stock use levels for bare ground within Dry communities, and species dispersion within Wet communities. Such results must be placed within the context of contemporary pack stock use records, which are maintained by the NPS. Our study did not account for past impacts from periods when pack stock use levels were much higher than what is currently allowed. For example, historical accounts of Sierra Club outings from the early 1900s have descriptions of over 100 pack animals being used on single trips to the Sierra Nevada high country [56]. With historically higher use levels, there is a possibility of legacy effects that are unaccounted for in the meadows we studied. While legacy effects could include altered hydrologic regimes, other legacies may encompass adaptation to grazing by meadow communities, shifts to a more resilient composition, as well as increased productivity [57]. There is also the possibility that the timing of our sampling in relation to concurrent grazing may have influenced our results. However, Cole et al. [15] found a within-year reduction in live plant biomass at any pack stock use level. Nevertheless, while such methodological limitations could have changed our data on vegetation cover and bare ground, it would not cause within-year shifts in species within these perennial dominated meadows.

Monitoring dynamic ecosystems is challenging, and any measure of change is dependent upon the variables monitored. We demonstrated that using a single variable indicator such as vegetation cover or bare ground could fail to distinguish other changes occurring within the meadow. However, regardless of the variable measured, no differences between stock and non-stock meadows would have been detected if the meadows we surveyed were not evaluated within the context of local hydrologic conditions.

## Supporting information

S1 Table Study locations. Geographical locations of study meadows. X, Y coordinates are meadow centroids projected in NAD 1983 UTM Zone 11N; Site refers to the National Park unit where each meadow is located; SampleYear refers to the year sampling occurred; matchGroup indicates the pairing results from the multivariate matching technique; Stock indicates whether a meadow was a pack stock meadow (1) or a non-stock meadow (0).
(CSV)

## References

[1] Graber D, 1996. Status of terrestrial vertebrates. In: Sierra Nevada Ecosystem Project: Final report to Congress. Vol II, chapter 27. University of California, Centers for Water and Wildland Resources, Davis, CA, US.

[2] Berlow EL, Knapp RA, Ostoja SM, Williams RJ, McKenny H, Matchett JR, et al A network extension of species occupancy models in a patchy environment applied to the Yosemite Toad (*Anaxyrus canorus*). PLoS One. 2013;8(8). 10.1371/journal.pone.0072200 PMC3741202 23951296

[3] Hammersmark CT, Rains MC, Mount JF. Quantifying the hydrological effects of stream restoration in a montane meadow, northern California USA. River Research and Applications. 2008; 24:735–753.

[4] National Park Service. General Management Plan (GMP)—Sequoia & Kings Canyon National Parks (U.S. National Park Service). Available from: https://www.nps.gov/seki/learn/management/gmp.htm

[5] McClaran MP, Cole DN. Packstock in wilderness: use, impacts, monitoring, and management. 1993;General Technical Report (September):33p.

[6] United States District Court Northern Distric for California: High Sierra Hikers Association (Plantiffs) v. United States Department of the Interior, National Park Service, Sequoia and Kings Canyon Natinoal Parks, Kenneth L. Salazar, No. CV-09-4621. Filed September 30, 2009

[7] Weixelman D, Hill B, Cooper D, Berlow E. A field key to meadow hydrogeomorphic types for the Sierra Nevada and southern Cascade Ranges in California. General Technical Report R5-TP-034. 2011.

[8] Wood S. Holocene stratigraphy and chronology of mountain meadows, Sierra Nevada, California. Ph.D. Dissertation. California Institute of Technology, Pasadena, California. 1975. Available from: http://thesis.library.caltech.edu/5570/1/Wood_sh_1975.pdf

[9] Allen-Diaz BH. Water table and plant species relationships in Sierra Nevada meadows. Am Midl Nat 1991;126(1):30–43.

[10] Lundquist JD, Cayan DR. Surface temperature patterns in complex terrain: daily variations and long-term change in the central Sierra Nevada, California. J Geophys Res Atmos. 2007;112(D11)

[11] Holmquist JG, Schmidt-Gengenbach J, Haultain SA. Effects of a long-term disturbance on arthropods and vegetation in subalpine wetlands: manifestations of pack stock grazing in early versus mid-season. PLoS One. 2013;8(1):1–10. 10.1371/journal.pone.0054109 PMC3538743 23308297

[12] Ostoja SM, Brooks ML, Moore PE, Berlow EL, Blank R, Roche J, et al Potential environmental effects of pack stock on meadow ecosystems of the Sierra Nevada, USA. The Rangeland Journal. 2014 p. 411–27.

[13] Fites-Kaufman J, Rundel P, Stephenson N, Weixelman D. Montane and subalpine vegetation of the Sierra Nevada and Cascade ranges. Terrestrial Vegetation of California. 2007. p. 456–501.

[14] McClaran MP. Recreational Pack Stock Management in Sequoia and Kings Canyon National Parks. Rangelands. 1989;11(1):3–8.

[15] van Wagtendonk JW, Parsons DJ. Wilderness Research and Management in the Sierra Nevada National Parks In: Halverson WL, Davis GE, editors. Sciecne Ecosystem Management in National Parks. Tucson, AZ: The University of Arizona Press; 1996 p. 281–294.

[16] Hopkinson P, Hammond M, Bartolome J, Brooks M, Berlow EL, Klinger R, et al. A natural resource condition assessment for Sequoia and Kings Canyon National Parks: Appendix 13- Meadows. Natural Resource Report NPS/SEKI/NRR-2013/665/13.National Park Service, Fort Collins, Colorado

[17] National Park Service. Wilderness Stewardship Plan (WSP)—Sequoia & Kings Canyuon National Parks (U.S. National Park Service). Available from: https://www.nps.gov/yose/getinvolved/wsp.htm

[18] Sekhon J. Multivariate and propensity score matching software with automated balance optimization: the matching package for R. J Stat Softw. 2011;42(1):1–52.

[19] R Core Team. R: A Language and Environment for Statistical Computing. Vienna, Austria; 2012.

[20] Legendre P, Legendre L. Numerical Ecology. 3rd ed. Elsevier; 2012.

[21] Ager AA, Owens KE. Charcterizating meadow vegetation with multitemporal Landsat thematic mapper remote sensing. Research Note, U.S. Department of Agriculture, Forest Service 2004

[22] Thornton PE, Thornton MM, Mayer BW, Wilhelmi N, Wei Y, Devarakonda R, et al. 2014. Daymet: Daily Surface Weather Data on a 1-km Grid for North America, Version 2. ORNL DAAC, Oak Ridge, Tennessee, USA. Time period: 1980-01-01 to 1997-12-31. http://dx.doi.org/10.3334/ORNLDAAC/1281

[23] National Park Service Integrated Resource Management Applications. Geospatial Datatsets (10m Digital Elevation Model; Geospatial Vegetation Information; Trails and Trailheads; Roads; Lakes). Available at irma.nps.gov

[24] Dozier J, Frew J. Computational provenance in hydrologic science: a snow mapping example. Philos Trans R Soc London A Math Phys Eng Sci 2009;367(1890):1021–33. 10.1098/rsta.2008.0187 19087938

[25] Crist EP, Cicone RC. A physically-based transformation of Thematic Mapper data—the TM tasseled cap. IEEE Transactions on Geoscience and Remote Sensing. 1984;GE-22(3):256–263.

[26] Landsat Thematic Mapper (TM). Data available from U.S. Geological Survey https://lta.cr.usgs.gov/TM

[27] Anderson GL, Hanson JD, Haas RH. Evaluating Landsat Thematic Mapper derived vegetation indices for estimating above-ground biomass on semiarid rangelands. Remote Sens. Environ. 1993;45:165–175.

[28] De’ath G, Fabricius KE. Classfication and regression tree: a powerful yet simple technique for ecological data analysis. Ecology. 2000;81(11):3178–92.

[29] Oksanen J, Blanchet FG, Kindt R, Legendre P, Minchin PR, O’Hara RB, et al. Vegan: community ecology package. 2012. Available from: http://cran.r-project.org/package=vegan

[30] Fraterrigo JM, Rusak JA. Disturbance-driven changes in the variability of ecological patterns and processes. Ecol Lett. 2008;11(7):756–70. doi: 10.1111/j.1461-0248.2008.01191.x 18422637 10.1111/j.1461-0248.2008.01191.x

[31] Anderson MJ. Distance-based tests for homogeneity of multivariate dispersions. Biometrics. 2006;62(1):245–53. doi: 10.1111/j.1541-0420.2005.00440.x 16542252 10.1111/j.1541-0420.2005.00440.x

[32] Ratliff RD. Meadows in the Sierra Nevada of California: state of knowledge. General Technical Report PSW-84. 1985.

[33] Graumlich LJ. A 1000-year record of temperature and precipitation in the Sierra Nevada. Quat Res. 1993;39(2):249–55.

[34] Laycock WA, Richardson BZ. Long-term effects of pocket gopher control on vegetation and soils of a subalpine grassland. J Range Manag. 1975;28(6):458–62.

[35] Cole DN, van Wagtendonk JW, McClaran MP, Moore PE, McDougald NK. Response of mountain meadows to grazing by recreational pack stock. J Range Manag. 2004;57(2):153–60.

[36] Vogel A, Scherer-Lorenzen M, Weigelt A. Grassland resistance and resilience after drought depends on management intensity and species richness. PLoS One. 2012;7(5):1–10. 10.1371/journal.pone.0036992 PMC3353960 22615865

[37] Beever EA, Brussard PF. Examining ecological consequences of feral horse grazing using exclosures. Western North American Naturalist. 2000; 60:236–254.

[38] Baccei JS, Hart SC, Mcclaran MP, Kuhn TJ. Multi-scale drivers of soil resistance in seasonally wet meadows of the Sierra Nevada Range, USA. Wetlands. 2017; In review.

[39] Kauffman JB, Krueger WC. Livestock impacts on riparian ecosystems and streamside management implications… a review. J Range Manag. 1984;37(5):430–8.

[40] Cluzeau D, Binet F, Vertes F, Simon JC, Riviere JM, Trehen P. Effects of intensive cattle trampling on soil-plant-earthworms system in two grassland types. Soil Biol Biochem. 1992;24(12):1661–5.

[41] Nadian H, Smith SE, Alston AM, Murray RS. Effects of soil compaction on plant growth phosphorus uptake and morphological characteristics of vesicular—arbuscular mycorrhizal colonization of *Trifolium subterraneum*. New Phytol. 1997;135(2):303–11.

[42] Tilman D, Reich PB, Knops JMH. Biodiversity and ecosystem stability in a decade-long grassland experiment. Nature. 2006 6 1;441(7093):629–32. doi: 10.1038/nature04742 16738658 10.1038/nature04742

[43] Belsky AJ, Matzke A, Uselman S. Survey of livestock influences on stream and riparian ecosystems in the western United States. J Soil Water Conserv. 1999;54(1):419–31.

[44] Weixelman DA, Zamudio DC, Zamudio KA, Tausch RJ. Classifying ecological types and evaluating site degradation. J Range Manag. 1997;315–21.

[45] Stringham TK, Krueger WC, Shaver PL. State and transition modeling: an ecological process approach. J Range Manag. 2003;56(2):106–13.

[46] Sasaki T, Okayasu T, Jamsran U, Takeuchi K. Threshold changes in vegetation along a grazing gradient in Mongolian rangelands. J Ecol. 2008;96(1):145–54.

[47] Kefi S, Rietkerk M, Alados CL, Pueyo Y, Papanastasis VP, Elaich A, et al Spatial vegetation patterns and imminent desertification in Mediterranean arid ecosystems. Nature. 2007 9 13;449(7159):213–7. doi: 10.1038/nature06111 17851524 10.1038/nature06111

[48] Dakos V, Carpenter SR, Brock WA, Ellison AM, Guttal V, Ives AR, et al Methods for detecting early warnings of critical transitions in time series illustrated using simulated ecological data. PLoS One. 2012;7(7):1–20. 10.1371/journal.pone.0041010 PMC3398887 22815897

[49] Collins SL, Smith MD. Scale-dependent interaction of fire and grazing on community heterogeneity in tallgrass prairie. Ecology 2006;87(8):2058–67. 16937645 10.1890/0012-9658(2006)87[2058:siofag]2.0.co;2

[50] Sterling A, Peco B, Casado MA, Galiano EF, Pineda FD. Influence of microtopography on floristic variation in the ecological succession in grassland. Oikos. 1984;42(3):334–42.

[51] Loheide SP, Booth EG. Effects of changing channel morphology on vegetation, groundwater, and soil moisture regimes in groundwater-dependent ecosystems. Geomorphology. 2011 3 15;126(3–4):364–76.

[52] del Moral R. Competition as a control mechanism in subalpine meadows. Am J Bot 1983;70(2):232–45.

[53] Diffenbaugh NS, Swain DL, Touma D. Anthropogenic warming has increased drought risk in California. PNAS 2015;112(13): 3931–3936. doi: 10.1073/pnas.1422385112 25733875 10.1073/pnas.1422385112 PMC4386330

[54] Berlow EL, D' Antonio CM, Swartz H. 2003. Response of herbs to shrub removal across natural and experimental variation in soil moisture. Ecological Applications 2003; 13:1375–1387.

[55] Griffin D, Anchkaitis KJ. How unusual is the 2012–2014 California drought? Geophyscial Research Letters 2014;41:9017–9023.

[56] McClaran MP. Recreational Pack Stock Management in Sequoia and Kings Canyon National Parks. Rangelands. 1989;11(1):3–8.

[57] Han JJ, Chen JQ, Han GD, Shao CL, Sun HL, Li LH. Legacy effects from historical grazing enhanced carbon sequestration in a desert steppe. Journal of Arid Environments. 2014; 107:1–9.

